# Survival of Patients with Sinonasal Cancers in a Population-Based Registry, Lombardy, Italy, 2008–2023

**DOI:** 10.3390/cancers16050896

**Published:** 2024-02-23

**Authors:** Dario Consonni, Simona Stella, Nerina Denaro, Alessandra Binazzi, Barbara Dallari, Sabrina Rugarli, Flavia Borello, Enzo Coviello, Carolina Mensi

**Affiliations:** 1 Occupational Health Unit, Fondazione IRCCS Ca’ Granda Ospedale Maggiore Policlinico, 20122 Milan, Italy; dario.consonni@policlinico.mi.it (D.C.); barbara.dallari@policlinico.mi.it (B.D.); sabrina.rugarli@policlinico.mi.it (S.R.); 2 Medical Oncology Unit, Fondazione IRCCS Ca’ Granda Ospedale Maggiore Policlinico, 20122 Milan, Italy; nerina.denaro@policlinico.mi.it; 3 Department of Occupational and Environmental Medicine, Epidemiology and Hygiene, National Institute for Insurance against Accidents at Work (INAIL), 00143 Rome, Italy; a.binazzi@inail.it; 4 SC Prevenzione e Sicurezza, ATS Milano Città Metropolitana, 20122 Milan, Italy; fborello@ats-milano.it; 5 Formerly at Epidemiology Service, Local Health Unit, 76123 Barletta, Italy; enzocovi@gmail.com

**Keywords:** sinonasal cancers, survival, occupational cancers, cancer registry, respiratory malignancy, net survival

## Abstract

**Simple Summary:**

Sinonasal cancers (SNCs) are rare tumours of the nasal cavity and paranasal sinuses with recognised or suspected associations with some occupational carcinogens, including wood and leather dusts and nickel and chromium compounds. In Italy, a population-based SNC registry organised as a network of regional registries was officially established in 2008 to monitor SNC occurrence and evaluate occupational exposure. In this study, we analysed survival of SNC patients based on the SNC registry of the Lombardy region, north-west Italy (10 million people, one-sixth of the Italian population). In 2008–2020, we recorded more than 800 cases. Vital status was determined until mid-2023. About 50% of patients died within 5 years of diagnosis. Survival was strongly dependent on patients’ age (worst prognosis in the elderly), tumour location (worst prognosis for paranasal sinuses), and for some histological types. Survival has not improved in recent years. Women and men had a similar prognosis.

**Abstract:**

Sinonasal cancers (SNCs) are rare malignancies associated with occupational exposures. The aim of this study was to analyse the survival of SNC patients using data from the population-based SNC registry of the Lombardy region (10 million people), Italy. We included epithelial SNC cases registered in 2008–2020 and followed-up for vital status until 31 July 2023. Multivariate flexible parametric models with time-dependent covariates were fitted to calculate excess hazard ratios (EHRs) and 95% confidence intervals (CIs) of death. Based on 827 cases (553 males, 274 females) and 514 deaths (345 males, 169 females), the 5-year observed survival was 49% and the net survival was 57%. Age had a substantial impact on survival, particularly within the first year (EHR, 1.35; 95% CI, 1.12–1.51 per 10 years). Compared with the nasal cavity, the EHR for paranasal sinuses was 4.70 (95% CI, 2.96–7.47) soon after diagnosis. Compared with squamous cell carcinomas, the EHR was 0.69 (95% CI, 0.52–0.91) for adenocarcinomas, 1.68 (95% CI, 1.20–2.35) for undifferentiated and unspecified carcinomas, and 1.78 (95% CI, 1.07–2.95) for neuroendocrine carcinomas. Age and cancer site showed time-dependent effects on prognosis, especially within the first month after diagnosis. Prognosis was also markedly affected by cancer morphology. No associations were found for gender and period of diagnosis.

## 1. Introduction

Sinonasal cancers (SNCs) are rare malignancies (<1% of all cancers) of the nasal cavity or paranasal sinuses (maxillary, ethmoidal, frontal, and sphenoidal) [1,2]. The SNC incidence rate is below 1–2 per 100,000 person-years and more frequent (roughly double) in men [3,4,5,6,7]. The most frequently affected anatomical sites are the nasal cavity and the maxillary and ethmoidal sinuses [3,4,5,6,7,8,9,10]. The majority of SNCs (80%) are epithelial; the most frequent morphologies are squamous cell carcinoma (SCC), mostly in the maxillary sinus and nasal cavity, and intestinal-type adenocarcinoma (ITAC), which often arises in the ethmoidal sinus [3,4,5,6,7,8,9,10].

Overall 5-year survival after SNC diagnosis is widely variable, depending on the cancer site, stage, histological type, and treatment [3,4,5,8,9,10]. Tumours with good/moderate histologic differentiation present a survival advantage over the poorly differentiated ones [8]. Because of the anatomic location, SNCs often have an unspecific onset of symptoms and are diagnosed at a late stage. They can produce significant morbidity resulting from the entanglement or encroachment of nearby structures (orbits, olfactory nerves, facial nerves, and the intracranial space). A review on outcomes of orbital exenteration (OE) in patients with craniofacial lesions (CFLs) showed that the most common lesion was SCC (31.8%) and the most common symptom was disturbed vision/reduced visual acuity (22.5%). Although OE is a disfiguring procedure with devastating outcomes, it is a viable option for carefully selected patients with advanced CFLs (5-year overall survival, 50%; median OS, 61 months) [10]. Surgery remains the mainstay of treatment (either endoscopic resection as the preferred or an external approach). However, even after radical surgery, overall survival rates at 3 and 5 years remain unsatisfactory (50–64% and 45–53%, respectively). The recurrence rate is about 20–30% and relapses are mainly local [9]. SNCs deserve a multidisciplinary approach in a tertiary centre (with a high volume of head and neck patients treated). Because of the rarity and morphological heterogeneity, evidence on therapeutic management is sparse, resulting in a lack of clinical guidelines [8].

The occurrence of epithelial SNCs is strongly associated with occupational exposures to several carcinogenic agents, with an attributable fraction of 20–46% overall and 77% for adenocarcinomas [11]. According to the International Agency for Research on Cancer (IARC), SNC carcinogens with sufficient evidence in humans include wood and leather dusts, nickel compounds, and tobacco smoking. Carcinogens with limited evidence include carpentry and joinery activities, chromium VI compounds, formaldehyde, and working in textile industries [12].

Due to the strong association of SNCs with exposure to occupational carcinogens, in Italy a specific nationwide population-based epidemiological surveillance system of SNC cases was implemented (law 81/2008) through a national SNC cancer registry (ReNaTuNS: Registro Nazionale Tumori Naso-Sinusali) [7,13]. The reporting of SNC cases to the national SNC registry is compulsory. The registry has a regional structure with local operating centres dedicated to the collection of SNC cases and to the assessment of previous exposure to occupational carcinogenic agents. In Italy, standardised SNC rates (standard: European population 2011) in men and in women in the period 2010–2014 were 0.59 and 0.24 cases per 100,000, respectively [7].

The Lombardy SNC Registry, established in 2008, covers a large population in a highly industrialised region in north-west Italy [14]. The aim of this study was to quantify the survival of SNC patients with a first diagnosis between 2008 and 2020 and with follow-up of vital status as of 31 July 2023 using data from the Lombardy SNC Registry. Observed and net survival were estimated, and several factors affecting prognosis were evaluated.

## 2. Methods

### 2.1. The Lombardy Sinonasal Cancer Registry

The Lombardy SNC Registry is a regional operating centre (Centro Operativo Regionale: COR)) of the national SNC cancer registry (ReNaTuNS) at the National Institute for Insurance Against Accidents at Work (INAIL) in Rome. It collects all primary epithelial malignant SNCs among residents in Lombardy (about 10 million inhabitants) [14]. SNC cases are reported to the registry by many departments involved in diagnosing and treating patients, including pathology, otolaryngology, maxillofacial surgery, and radiotherapy wards. Since compulsory reporting is far from satisfactory, the completeness of the SNC case collection is achieved by periodic linkages with various databases (including pathology databases, hospital discharge databases, and mortality registries). After a careful review of medical records, an SNC diagnosis is classified following the national registry guidelines as “definite” (with histological confirmation) or “probable” (usually a diagnosis through computer tomography or magnetic resonance imaging) [14].

For each confirmed SNC case, information is collected through a standardised questionnaire administered by trained interviewers to the patient or, in cases of death or serious illness, to the next of kin. The questionnaire includes detailed sections on lifetime occupational history (including industrial sectors, jobs, and tasks performed) and extra-occupational exposures (including residential information, hobbies, and lifestyle). Exposure to known/suspected carcinogens is evaluated by a panel of experts and classified according to national guidelines. Information on previous cancers (at any site), head and neck radiotherapy, sinonasal diseases, and oestrogen therapy is also collected.

### 2.2. Statistical Analysis

Records of SNC cases first diagnosed between 2008 and 2020 were extracted from the Lombardy SNC Registry database. Follow-up of vital status was current as of 31 July 2023. Cancer site was classified according to the codes of the International Classification of Diseases, Tenth Revision (ICD-10) as follows: C30.0 (nasal cavity), C31.0 (maxillary sinus), C31.1 (ethmoidal sinus), C31.2 (frontal sinus), C31.3 (sphenoidal sinus), and C31.8 (overlapping sites of accessory sinuses). Histological types were coded according to the International Classification of Diseases for Oncology, Third Edition (ICD-O-3) and grouped according to the 2017 World Health Organization (WHO) Classification into the following groups: squamous cell carcinoma and variants, adenocarcinoma, neuroendocrine carcinoma, other epithelial neoplasms (including sinonasal undifferentiated carcinomas (SNUCs) and not-otherwise-specified (NOS) carcinoma), and malignant tumours [15].

Kaplan–Meier analysis was conducted to estimate 1-year, 3-year, and 5-year observed overall survival, which depends on survival associated with SNCs as well as with other diseases. Net survival was calculated using the Pohar Perme method to quantify survival exclusively due to SNCs [16]. The estimation of net survival requires the calculation of expected survival (i.e., survival relative to a comparable population group from the general population), for which we used Lombardy mortality tables (2008–2022) stratified by calendar year, gender, and age (1-year categories) downloaded from the website of the National Institute of Statistics (ISTAT) [17].

Univariate and multivariable flexible parametric excess hazard models [18] were fitted to estimate excess hazard ratios (EHRs) and 95% confidence intervals (CIs) of death adjusted for gender, age (0–49, 50–69, and 70+ years), period of diagnosis (2008–2012, 2013–2016, and 2017–2020), cancer site (nasal cavity vs. paranasal sinuses), and cancer morphology (squamous cell carcinoma and variants, adenocarcinoma, neuroendocrine carcinoma, and other morphologies including undifferentiated and unspecified carcinomas). EHR proportionality was assessed by likelihood-ratio tests (LRTs) to evaluate potential time-dependent effects. Data management and statistical analyses were performed using Stata 18 [19].

## 3. Results

### 3.1. Patient Characteristics

We included 827 patients with SNCs registered from 2008 to 2020 (553 males and 274 females; male–female ratio, 2:1), with a median age of 68–69 years, and equally distributed by gender over periods of diagnosis (Table 1). The most common cancer site was the nasal cavity, followed by maxillary (more common in females) and ethmoidal (more common in males) sinuses. In more than one-fourth of patients, the tumour involved multiple paranasal sinuses. Squamous cell carcinomas and variants were slightly more frequent in women, while adenocarcinomas were more frequent in men. Men were more frequently cigarette smokers (former or current) than women and were more frequently exposed to carcinogenic agents (39.2% vs. 13.1%), particularly wood and leather dusts. A total of 78 (14.1%) males and 29 (10.6%) females were patients with a previous cancer (Supplementary Table S1). Few underwent previous head and neck radiotherapy for other cancers. Various proportions of patients with SNCs had other sinonasal diseases (mostly rhinosinusitis), and about one-fourth of the women had used oestrogens.

### 3.2. Survival Analysis

**Figure 1:**
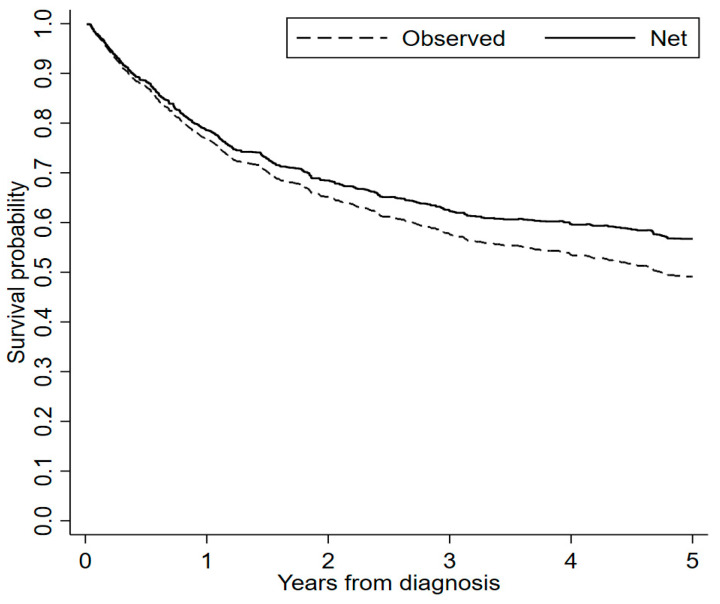
Overall observed and net survival curves of patients with sinonasal cancer (SNC), Lombardy SNC Registry, 2008–2020. Follow-up, 2008–2023.

As of 31 July 2023, we recorded 345 deaths out of 553 men (62.4%) and 169 deaths out of 274 women (61.7%). As expected, net survival was higher than observed survival (Figure 1). Five years after diagnosis, observed and net survival were 49% (95% CI, 46–53) and 57% (95% CI, 53–61), respectively.

Observed survival was similar in men and women and across the period of diagnosis but much lower in elderly patients (70+ years at diagnosis), in patients with SNC of paranasal sinuses, and in patients with neuroendocrine carcinoma and other carcinomas (undifferentiated and unspecified) (Table 2). Similar patterns were found with net survival (Table 3).

Table 4 reports EHRs of death calculated using univariate and multivariate flexible parametric hazard models with time-dependent effects of age and cancer site. Gender and period of diagnosis were weakly associated with prognosis, while age showed an effect particularly during the first month after diagnosis (adjusted EHR, 1.35 per 10-year increase). Paranasal cancers had the highest impact on prognosis soon after diagnosis (adjusted EHR, 4.70) and a slowly decreasing effect over time. Compared with squamous cell carcinomas and variants, we estimated a lower risk of death for adenocarcinomas (adjusted EHR, 0.69) and increased risks for neuroendocrine carcinoma (adjusted EHR, 1.78) and other epithelial neoplasms (adjusted HER, 1.68). In Table 4, for completeness, we report the fixed effect of age (HER, 1.05) and paranasal cancer site (HER, 2.46). However, they represent “residual” effects in a model with time-dependent variables; therefore, they should not be considered in the interpretation of results.

**Figure 2:**
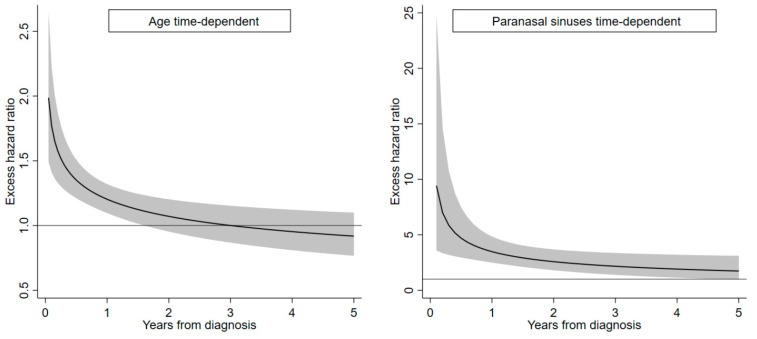
Time-dependent excess hazard ratios (EHRs) of death and 95% confidence bands (grey areas) for age (continuous, 10 years) and cancer of the paranasal sinuses (vs. nasal cavity), Lombardy SNC Registry, 2008–2020. Follow-up, 2008–2023. Estimates calculated from a multivariable flexible parametric model adjusted for gender, age (time-dependent), period of diagnosis, cancer site (time-dependent), and cancer morphology.

To visualise the time pattern of age and cancer site, we plotted two curves in which time-dependent effects are considered as continuous, not at fixed time points (0.5, 2.5, and 4.5 years) as in Table 4. The time-dependent effects of age are appreciable until about 2 years from diagnosis, while the effect of cancer site is evident up to 5 years (Figure 2).

## 4. Discussion

In this study, we estimated that the observed survival 5 years after diagnosis was 49%, while, as expected, the net survival (i.e., the survival specifically associated with SNCs relative to a similar population) was higher (57%). Survival was not or weakly associated with gender and period of diagnosis but strongly dependent on age, cancer site, and morphology. In addition, we documented time-dependent patterns (higher mortality soon after diagnosis) for age and paranasal sinuses.

Regarding morphology, we performed preliminary survival analyses by subdividing squamous cell carcinomas into keratinising, non-keratinising, and other squamous cell carcinomas, while adenocarcinomas were subdivided into ITAC, adenoid cystic, and other adenocarcinomas. Since the number of cases in these categories was small and/or survival did not differ much, we used the above-mentioned grouping in four groups.

With regard to tobacco smoking and occupational exposure, a priori we did not expect them to affect SNC patients’ survival. Moreover, the calculation of net survival can be questionable because the mortality tables were not stratified by these variables. We also note that none of the previous studies considered these variables in survival analyses. However, since some readers might be interested in these results, we performed crude analyses by calculating crude EHRs for these variables. The HERs for former and current smokers were 0.98 (95% CI, 0.72–1.34) and 1.13 (95% CI, 0.84–1.52), respectively. The HER for occupational exposure to carcinogens (ever vs. never exposed) was 0.91 (95% CI, 0.68–1.21). These results confirmed our expectation. For these reasons, we did not include these variables in the multivariable model.

### 4.1. Strengths and Limitations

Our study has several strengths. First, as it is based on a population-based registry and not on a selected case series, we can present a reliable picture of survival in the general population over a long period of observation. Second, this is a virtually complete case collection with good-quality information on SNC diagnosis. Third, beyond the classical estimation of observed survival, we applied a relative-survival approach that allowed us to consider all causes of death different from SNC. The estimation of relative or net survival avoids the need for information on cause of death, which is not always available in registry data; moreover, compared with cause-specific mortality, relative or net survival captures mortality associated with SNC even in cases in which the death certificate reports other causes of death (e.g., death due to cardiovascular complications or side effects of therapeutic interventions) [16,20]. The assumption is that patients in the study are similar to the general population; for this reason, we used year-, gender-, and age-specific life tables of the same population of SNC cases over the same time period. In addition, we applied multiple flexible parametric excess hazard models to evaluate fixed and time-dependent effects [18].

This study has also some weaknesses. First, a limitation that affects population-based registries in general is the lack of data on tumour stage, on therapeutic interventions, and on disease biomarkers that are used by oncologists to inform therapeutic options. Second, since these cancers are particularly aggressive, is not always possible to interview the patients because of serious medical conditions or emotional difficulties. In fact, in a sizable proportion of patients, the interview has to be conducted with the next of kin, who may be ignorant of important information, particularly regarding the occupational history of the SNC patient.

### 4.2. Comparison with Other Studies

Our estimated 5-year observed and net survival are slightly lower but not much different from those reported in studies based on data from population-based SNC series in Denmark and the USA [3,4,5]. We found no observed or net survival differences in males and females, consistently with one of the US studies that examined 6739 SNC patients over a long time period (1973–2006) [3]. The other study, on 13,295 patients examined in 1983–2011, unfortunately did not report survival results stratified by gender [5]. Conversely, the Danish study (1720 patients studied during the period 1980–2014) estimated a lower 5-year relative survival rate in males (52%) compared with females (58%), while the 10-year survival rate was similar (40% in men, 42% in women) [4]. In these three studies, no analysis of survival by age was performed, while, in general, our results regarding survival according to cancer site and morphology are consistent with theirs [3,4,5]. The close relation between prognosis and histology was confirmed in a recent multicentre study including a series of European patients [21].

The higher relative survival for nasal cavity compared with maxillary sinus may be due to the earlier diagnosis of tumours in the nasal cavity because of the earlier detection of symptoms (e.g., unilateral nasal obstruction or epistaxis) and easier surgical access [22]. We found that the poorest prognosis was for patients with neuroendocrine carcinomas and other epithelial neoplasms, including undifferentiated carcinomas (SNUC) and unspecified carcinomas. The poor prognosis for undifferentiated carcinomas was reported by others [1,23]. Neuroendocrine carcinomas are rare and have a broad spectrum of histological differentiations and behaviours due to the very aggressive nature of poorly differentiated (small and non-small) cell types. A multidisciplinary therapeutic approach is required, and radiation therapy followed by chemotherapy could be preferred to mutilating surgery as a first approach [24].

Finally, although not strictly relevant to the aim of this work, we note that information on potential risk factors for SNC occurrence was collected for a high percentage of patients (87.7% of males and 81.4% of females) through interviews with a standardised instrument. We could therefore document exposure to occupational carcinogens (the main focus of the national SNC registry) for a sizable percentage of patients (mainly men). This is in agreement with national data and other studies [7,25,26,27]. These findings support the important role of SNC surveillance through a dedicated registry to increase awareness of the occupational risks for this disease and to inform policies for prevention and for compensation of affected workers.

## 5. Conclusions

This study was based on virtually complete and good-quality data from a population-based registry covering 10 million people over a long time. We confirmed that the survival of patients with sinonasal cancer strongly depended on age and cancer site and morphology. In addition, we documented marked time-dependent patterns (higher mortality soon after diagnosis) for age and cancer of the paranasal sinuses. Males and females showed a similar prognosis. We did not find a better prognosis in patients diagnosed more recently. This may suggest that the results of therapeutic efforts, which have been improving over time, are not yet appreciable, at least at the population level.

## Figures and Tables

**Table 1:** Characteristics of patients with sinonasal cancer by gender, Lombardy SNC Registry, 2008–2020.

Variable	Males		Females		*p*-Value ^a^
	No.	%	No.	%	
No. of subjects	553	100.0	274	100.0	
Age (median, min–max)	69.0	25.7–93.1	68.3	21.1–99.7	0.18
Period of diagnosis					
2008–2012	215	38.9	109	39.8	0.97
2013–2016	159	28.8	77	28.1	
2017–2020	179	32.4	88	32.1	
Cancer site (ICD-10 code)					
Nasal cavity (C30.0)	215	38.9	99	36.1	0.01
Maxillary sinus (C31.0)	95	17.2	72	26.3	
Ethmoidal sinus (C31.1)	73	13.2	21	7.7	
Frontal sinus (C31.2)	2	0.4	3	1.1	
Sphenoidal sinus (C31.3)	13	2.4	11	4.0	
Multiple sites (C31.8)	155	28.1	68	24.8	
Cancer morphology					
Squamous cell carcinoma and variants	255	46.1	145	52.9	0.03
Adenocarcinoma	214	38.7	78	28.5	
Neuroendocrine carcinoma	19	3.4	7	2.6	
Other epithelial neoplasm ^b^	55	10.0	32	11.7	
Malignant tumour	2	0.4	2	0.7	
Unknown ^c^	8	1.5	10	3.7	
Interview					
Patient	352	63.7	159	58.0	0.05
Next of kin	133	24.1	64	23.4	
Not performed	68	12.3	51	18.6	
Cigarette smoking					
Never	154	27.9	122	44.5	<0.001
Former	207	37.4	55	20.1	
Current	162	29.3	74	27.0	
Unknown	30	5.4	23	8.4	
Occupational exposure					
Never exposed	268	48.5	187	68.3	
Ever exposed (any agent)	217	39.2	36	13.1	<0.001
Wood dusts	150	27.1	13	4.7	<0.001
Leather dusts	65	11.8	20	7.3	0.01
Nickel compounds	9	1.6	3	1.1	0.05
Chromium VI compounds	18	3.3	3	1.1	0.01
Formaldehyde	3	0.5	1	0.4	0.05
Unknown	68	12.3	51	18.6	<0.001

ICD-10, International Classification of Diseases, Tenth Edition. ^a^ From a chi-squared test, except for age (Wilcoxon rank-sum test). ^b^ Sinonasal undifferentiated carcinoma (SNUC) and not otherwise specified (NOS) carcinoma. ^c^ SNC cases with diagnostic certainty “probable” and without biopsy.

**Table 2:** Overall 1-year, 3-year, and 5-year observed survival (%) of patients with sinonasal cancer according to selected risk factors, Lombardy SNC registry, 2008–2020. Follow-up, 2008–2023.

Variable	1-Year (95% CI)	3-Year (95% CI)	5-Year (95% CI)
All patients	77 (74–79)	58 (54–61)	49 (46–53)
Gender			
Male	77 (73–80)	57 (53–61)	49 (45–54)
Female	76 (71–81)	59 (53–64)	49 (43–55)
Age			
0–49	85 (77–90)	67 (57–74)	59 (49–67)
50–69	85 (81–89)	69 (64–74)	62 (57–67)
70+	68 (63–72)	46 (41–51)	36 (31–41)
Period of diagnosis			
2008–2012	76 (71–81)	58 (52–63)	50 (44–55)
2013–2016	77 (71–82)	56 (50–62)	47 (40–53)
2017–2020	77 (72–82)	59 (53–65)	51 (45–57)
Cancer site (ICD-10 code)			
Nasal cavity (C30.0)	79 (72–83)	64 (58–69)	57 (51–62)
Paranasal sinuses (C31)	75 (71–79)	54 (50–58)	44 (40–49)
Cancer morphology			
Squamous cell carcinoma and variants	79 (74–82)	61 (56–66)	53 (47–57)
Adenocarcinoma	82 (77–86)	65 (59–70)	54 (48–59)
Neuroendocrine carcinoma	73 (52–86)	31 (15–49)	27 (12–44)
Other epithelial neoplasm ^a^	63 (62–72)	39 (29–49)	37 (27–47)

CI, confidence interval; ICD-10, International Classification of Diseases, Tenth Edition. ^a^ Sinonasal undifferentiated carcinoma (SNUC) and not-otherwise-specified (NOS) carcinoma.

**Table 3:** Overall 1-year, 3-year, and 5-year net survival (%) of patients with sinonasal cancer according to selected risk factors, Lombardy SNC registry, 2008–2020. Follow-up, 2008–2023.

Variable	1-Year (95% CI) ^a^	3-Year (95% CI) ^a^	5-Year (95% CI) ^a^
All patients	79 (76–82)	62 (59–66)	57 (53–61)
Gender			
Male	79 (76–83)	63 (59–68)	59 (54–64)
Female	77 (72–83)	62 (56–68)	53 (47–60)
Age			
0–49	85 (78–92)	67 (59–76)	59 (51–69)
50–69	86 (82–90)	71 (66–76)	65 (60–71)
70+	71 (66–76)	55 (49–61)	50 (43–57)
Period of diagnosis			
2008–2012	78 (73–83)	62 (56–68)	57 (51–63)
2013–2016	79 (73–85)	61 (55–69)	55 (48–64)
2017–2020	79 (74–84)	64 (58–71)	58 (51–67)
Cancer site (ICD-10 code)			
Nasal cavity (C30.0)	81 (76–86)	69 (63–75)	65 (58–72)
Paranasal sinuses (C31)	77 (73–81)	59 (54–64)	52 (47–58)
Cancer morphology			
Squamous cell carcinoma and variants	80 (76–85)	65 (60–71)	60 (54–66)
Adenocarcinoma	84 (80–89)	71 (65–77)	64 (57–71)
Neuroendocrine carcinoma	74 (59–93)	32 (19–56)	29 (16–54)
Other epithelial neoplasm ^b^	64 (55–75)	41 (31–53)	39 (29–51)

CI, confidence interval; ICD-10, International Classification of Diseases, Tenth Edition. ^a^ From Pohar Perme estimation. ^b^ Sinonasal undifferentiated carcinoma (SNUC) and not-otherwise-specified (NOS) carcinoma.

**Table 4:** Excess hazard ratios (EHRs) of death according to selected risk factors, Lombardy SNC Registry, 2008–2020. Follow-up, 2008–2023.

Variable	Deaths N (%)	Crude EHR	95% CI	Adjusted EHR ^a^	95% CI
Gender					
Female	169 (61.7)	1.00	Reference	1.00	Reference
Male	345 (62.4)	0.91	0.71–1.15	0.94	0.74–1.20
Age (continuous, 10 years)		1.00	0.89–1.13	1.05	0.93–1.19
0.5 years from diagnosis		1.32	1.19–1.47	1.35	1.12–1.51
2.5 years from diagnosis		0.97	0.86–1.10	1.03	0.90–1.17
4.5 years from diagnosis		0.87	0.73–1.02	0.93	0.78–1.11
Period of diagnosis					
2008–2012	237 (73.2)	1.00	Reference	1.00	Reference
2013–2016	150 (63.6)	1.02	0.77–1.35	1.06	0.79–1.41
2017–2020	127 (47.6)	0.89	0.67–1.18	0.97	0.73–1.28
Cancer site (ICD-10 code)					
Nasal cavity (C30.0)	152 (48.4)	1.00	Reference	1.00	Reference
Paranasal sinuses (C31)	362 (70.6)	3.32	2.43–4.53	2.46	1.69–3.58
0.5 years from diagnosis		4.48	2.85–7.03	4.70	2.96–7.47
2.5 years from diagnosis		2.37	1.61–3.51	2.34	1.57–3.48
4.5 years from diagnosis		1.88	1.10–3.21	1.81	1.05–3.14
Cancer morphology					
Squamous cell carcinoma and variants	234 (58.5)	1.00	Reference	1.00	Reference
Adenocarcinoma	176 (60.3)	0.86	0.65–1.14	0.69	0.52–0.91
Neuroendocrine carcinoma	23 (88.5)	2.23	1.35–3.69	1.78	1.07–2.95
Other epithelial neoplasm ^b^	60 (69.0)	1.94	1.38–2.71	1.68	1.20–2.35

CI, confidence interval; EHR, excess hazard ratio; ICD-10, International Classification of Diseases, Tenth Edition. ^a^ From a flexible parametric excess hazard model containing all the covariates shown in the table. ^b^ Sinonasal undifferentiated carcinoma (SNUC) and not-otherwise-specified (NOS) carcinoma.

## Data Availability

Data presented in this study are available on request from the corresponding author.

## Supplementary Materials

The following supporting information can be downloaded at: https://www.mdpi.com/article/10.3390/cancers16050896/s1. Table S1. Previous malignant tumours (any site), head and neck radiotherapy for other diseases, sinonasal diseases, and oestrogen therapy in patients with sinonasal cancer (SNC) by gender; Lombardy SNC Registry, 2008–2020.
