# Supplementary material for: Survival of Patients with Sinonasal Cancers in a Population-Based Registry, Lombardy, Italy, 2008–2023
Source: Cancers (Basel). 2024 Feb 23;16(5):896. doi: 10.3390/cancers16050896 (PMC10930825; doi:10.3390/cancers16050896)
Supplement: Supplementary file 1 [file cancers-16-00896-s001.zip › cancers-2855717-supplementary.pdf]

Supplementary Table S1. Previous malignant tumours (any site), head and neck radiotherapy for other diseases, sinonasal diseases, and oestrogen therapy in patients with sinonasal cancer (SNC) by gender; Lombardy SNC Registry, 2008-2020.

**Variable**	**Males**		**Females**		**P-value** [a]
	**No.**	**%**	**No**	**%**	
Malignant tumour					0.31
No	405	73.2	205	74.8	
Yes	78	14.1	29	10.6	
Unknown	70	12.7	40	14.6	
Radiotherapy					
No	489	88.4	224	81.8	0.01
Yes	11	2.0	3	1.1	
Unknown	53	9.6	47	17.2	
Polyps					
No	469	84.8	211	77.0	0.01
Yes	34	6.2	18	6.6	
Unknown	50	9.0	45	16.4	
Hypertrophy of nasal turbinates					
No	476	86.1	215	78.5	0.02
Yes	23	4.2	17	6.2	
Unknown	54	9.8	42	15.3	
Allergic rhinitis					
No	458	82.8	199	72.6	<0.001
Yes	40	7.2	30	11.0	
Unknown	55	10.0	45	16.4	
Rhinosinusitis					
No	418	75.6	183	66.8	0.01
Yes	82	14.8	47	17.2	
Unknown	53	9.6	44	16.1	
Deviated septum					
No	408	73.8	200	73.0	<0.001
Yes	94	17.0	28	10.2	
Unknown	51	9.2	46	16.8	
Malignant tumour					0.31
No	405	73.2	205	74.8	
Yes	78	14.1	29	10.6	
Unknown	70	12.7	40	14.6	
Radiotherapy					
No	489	88.4	224	81.8	0.01
Yes	11	2.0	3	1.1	
Unknown	53	9.6	47	17.2	
Oestrogen therapy					
No	511	92.4	154	56.2	<0.001
Yes	0	0	74	27.0	
Unknown	42	7.6	46	16.8	

[a] From chi-squared test.
